# A Thematic Analysis of Stigma and Disclosure for Perinatal Depression on an Online Forum

**DOI:** 10.2196/mental.5611

**Published:** 2016-05-19

**Authors:** Donna Moore, Susan Ayers, Nicholas Drey

**Affiliations:** ^1^ Centre for Maternal and Child Health School of Health Sciences City University London London United Kingdom; ^2^ Centre for Public Health Research School of Health Sciences City University London London United Kingdom

**Keywords:** perinatal, online, Internet, depression, eHealth

## Abstract

**Background:**

Perinatal mental illness is a global health concern; however, many women do not get the treatment they need to recover. Some women choose not to seek professional help and get no treatment because they feel stigmatized. Online forums for various health conditions, including perinatal mental health, can be beneficial for members. Little is known about the role that online forums for perinatal mental illness play in reducing stigma and subsequent disclosure of symptoms to health care providers and treatment uptake.

**Objective:**

This study aimed to examine stigma and disclosure in forums and describe any potential disadvantages of forum use.

**Methods:**

An online forum for mothers was examined and 1546 messages extracted from 102 threads from the antenatal and postnatal depression section. These messages were subjected to deductive systematic thematic analysis to identify common themes regarding stigma and disclosure of symptoms and potential disadvantages of forum use.

**Results:**

Two major themes were identified: *stigma* and *negative experiences of disclosure*. Stigma had 3 subthemes: internal stigma, external stigma, and treatment stigma. Many women were concerned about feeling like a “bad” or “failed” mother and worried that if they disclosed their symptoms to a health care provider they would be stigmatized. Posts in response to this frequently encouraged women to disclose their symptoms to health care providers and accept professional treatment. Forum discourse reconstructed the ideology of motherhood as compatible with perinatal mental illness, especially if the woman sought help and adhered to treatment. Many women overcame stigma and replied that they had taken advice and disclosed to a health care provider and/or taken treatment.

**Conclusions:**

Forum use may increase women's disclosure to health care providers by challenging their internal and external stigma and this may strengthen professional treatment uptake and adherence. However, a few posts described negative experiences when disclosing to health care providers.

## Introduction

Perinatal mental illness is a global health concern and includes antenatal and postnatal depression and anxiety disorders, post-traumatic stress disorder after childbirth, and adjustment disorders. Perinatal depression has a prevalence of 12%-20% [1] and perinatal anxiety affects 2.6%-39% of women [2]. Post-traumatic stress disorder after childbirth affects 3.17% of new mothers and 15% in high-risk groups [3]. If these illnesses are not treated, there are well-documented adverse outcomes for women, infants, and families [4]. Detrimental maternal outcomes include substance abuse and suicide [5] and untreated antenatal depression is associated with postnatal depression [6]. Negative infant outcomes include developmental and cognitive delays [7,8], preterm delivery [9], and an increased risk of behavioral and attachment problems [10].

Women with perinatal mental illness often fail to receive treatment despite treatment being available [11]. Often this is because women choose not to disclose and seek help from their health care provider. Poor knowledge of medical conditions is one reason some people may be less likely to recognize they are ill and therefore less likely to seek help [12]. Some women have poor health literacy about perinatal mental illness and problems relating professional health information to how they experience the illness [13]. In addition, there can be a misconception about what perinatal mental illness is, and women may find it difficult to distinguish what is a healthy emotional reaction to the transition to motherhood and what emotions may indicate a mental illness [14]. These issues can contribute to stigma.

Stigma is an extreme disapproval of someone or group of people because of a certain characteristic; it can present as external stigma where the general public holds a stigmatizing attitude. It can also present as internal stigma where the stigmatized individual believes this negative appraisal and applies it to themselves. There are high levels of external stigma in the general population and internal stigma has been identified in approximately a third of people with severe mental illnesses [15,16]. There are well-documented negative outcomes for individuals with mental illness because of external stigma such as social exclusion, discrimination, and fewer life opportunities [17]. Similarly, internal stigma has been associated with low self-esteem, reduced disclosure, and reluctance to seek treatment [17,18].

Thus, some women may think others will view them negatively for having a mental illness (external stigma) and may also feel bad about themselves for having a mental illness (internal stigma). Stigma is a major barrier to disclosure and help-seeking in the perinatal period [14,19]. Some women feel stigmatized not only because they have a mental illness but principally because they are a mother with a mental illness. This two-fold stigma means they are concerned about feeling like, and being seen by others as, a “bad mother” [20]. Women with perinatal mental illness have unique concerns related to their maternal identity; they may worry that having a mental illness would result in negative consequences such as social services involvement, loss of custodial rights, and hospitalization [21-23]. These features of stigma can contribute to reluctance to disclose symptoms [24]. A systematic review and meta-synthesis identified stigma and concerns about child custody as a key barrier to care for postnatal women [25]. Many women avoided disclosing symptoms to health care providers, as they did not want to be diagnosed as having a mental illness. They wanted to be seen as coping and were worried they would lose custody of their child should they disclose.

People suffering from stigmatized illnesses are more likely to turn to the Internet for help [26,27]. Studies have detailed the benefits of using online social support for a variety of health issues [28,29]. The Internet could provide a unique avenue to reduce stigma in terms of knowledge and attitude. It could provide information about perinatal mental illness that women could relate to. This could aid disclosure by increasing women's health literacy about perinatal mental illness and enable them to recognize that they have a problem.

There are thousands of websites dedicated to perinatal mental health and many online support groups or forums, but little is known about how members engage with them [30,31]. A content analysis by Evans et al [32] of an online support group for postnatal depression reported it was nonjudgmental. The forum provided emotional, informational, and some instrumental support. Posts encouraged users to contact a health care provider and take medication; there were no posts containing negative experiences with health care providers. Similarly, another content analysis documented how online support forums for lesbians with postnatal depression provided social support [33]. Many women were reluctant to disclose and seek help because of the stigma of being seen as an unfit mother and fear of the child being taken away. In addition to the stigma around their mental illness, women felt stigmatized because they were homosexual. The dichotomy of “good mother” “bad mother” deterred help-seeking behavior; it is plausible that this may be because it increases internal and external stigma. To some women the idea of a “good mother” is not compatible with mental illness; similarly, having symptoms of perinatal mental illness such as bonding problems or thinking of harming your child can make women feel like a “bad mother” [20].

A discourse analysis of an online forum for postnatal depression reported how it provided a place for mothers to confess their shame about having perinatal mental illness [34]. This enabled many women to overcome the stigma of being mentally ill and not meeting the expectations of a good mother. Women found that no one talked about this offline, so they constructed an online dialogue with other forum users that expressed negative feelings around motherhood. Nevertheless, it is important to note that some recent research suggests online support groups may not challenge stigma enough to affect or change help-seeking behavior [35]. Excessive participation in online support groups could also be a form of social avoidance and prevent disclosure and foster over reliance on forums [36]. Internet forums for perinatal mental health have yet to be researched to see how using forums may increase disclosure of symptoms and help-seeking behavior. Forums may challenge stigma by providing a unique source of experiential information and a space for women to disclose and seek advice anonymously without fear of being stigmatized. Posts on forums may challenge stigma and provide positive discourse about perinatal mental health. Posts may also provide encouragement for women to seek and adhere to professional treatment by challenging external stigma.

This study aimed to investigate if and how perinatal mental illness forums might overcome the barriers stigma presents to some women with perinatal mental illness when seeking help from health care providers and to see if there are possible disadvantages of using forums regarding disclosure and stigma.

## Methods

### Sample

Forums were identified using the three most popular UK search engines (Google, Bing, and Yahoo) that are used by 98.83% of Web users [37]. The text searches were “postnatal depression,” “postnatal forum,” “postnatal anxiety,” and “birth trauma” and entered into each of the search engines. The first 25 websites and their hyperlinks were assessed for inclusion in the study. Inclusion criteria were as follows: (1) they had a forum or message board dedicated to antenatal and/or postnatal mental health, (2) they had been active for the last 6 months, (3) the forum had more than 50 members, (4) messages could be viewed by nonmembers of the group, and (5) moderators gave permission to research their forums. Nine forums were contacted but only 1 forum moderator gave permission to research posts. The forum moderator was from “Mumsnet,” one of the largest websites for parenting advice and has active forums with between 1.2 and 1.7 million members [38]. There were 28 “talk topics” that contained between 1 and 273 forums. The forum section for antenatal and postnatal depression was dedicated to perinatal mental health and was used to draw the data for analysis.

### Procedure

Nineteen forum moderators were contacted and written permission from 1 moderator was obtained. Visitors were informed of the nature of the research and their right to withdraw their data via a prominent disclaimer on the forum. A link from the site provided details about what data were taken from the site and how the information was used. The study was retrospective to avoid influencing the participants’ interactions. Confidentiality was maximized by ensuring the anonymity of participants by replacing their user names with pseudo names.

### Data Selection

All messages on the antenatal and postnatal forum between January 2013 and June 2013 were included for analysis. This comprised 1546 messages retrieved from 102 threads. The average number of posts in a thread was nearly 28. These threads and messages were copied into Microsoft Word files and stored securely for data protection purposes and because forums can terminate at any time.

### Ethical Considerations

The study received ethical approval from the School of Health Sciences Research Ethics Committee, City University London. Precautions were taken to ensure the safety, dignity, and rights of participants in accordance with the 2007 “Guidelines for Ethical Practice in Psychological Research Online” as outlined by the British Psychological Society [39]. Consideration was given to the nature of online private and public spaces, anonymity, confidentiality, valid consent, and the right to withdraw from the study [40].

### Analysis

Discussion threads were examined using deductive systematic thematic analysis from a realist stance [41]. Threads were copied into the qualitative data analysis computer software NVivo 10 and threads were read and reread before generating initial codes [42]. Themes were generated from patterns in the codes and were included when they were frequent, appeared important to posters, and were related to the research aims. The principal researcher had experience in qualitative analyses and met regularly with a senior health researcher (SA) to discuss analysis, thus increasing reliability of codes and themes. The whole dataset was recoded when themes were defined and codes were organized to address the research questions of stigma, disclosure, and messages that could potentially hinder women seeking help. The principal researcher developed the interpretation of themes and final interpretations were agreed by consensus of all authors.

## Results

Two major themes were identified: stigma and negative experiences. Stigma had 3 subthemes: internal stigma, external stigma, and treatment stigma.

### Stigma

The majority of women disclosed their symptoms on their first post and often sought advice on diagnosis, whether or not they should contact a health care provider, healthcare providers’ attitudes to illness and treatment. Nearly all the replying posts urged women to contact their healthcare providers and often reassured women who had concerns about approaching health care providers. Women were frequently encouraged to honestly disclose their symptoms to health care providers and take professional treatment offered.

### Internal Stigma

Internal stigma was coded when women wrote about their stigmatized attitudes towards themselves, such as feelings of inadequacy as a mother. Many women used the forum to disclose shameful feelings often hidden from others such as feeling like a failure as a mother, wanting to leave the baby or family, intrusive thoughts of self-harm and child-abuse. They felt that there was no place offline to talk about the negative side of pregnancy and motherhood and valued the nonjudgmental space offered by forums. Replies were often reassuring and challenged internal stigma by stressing that these feelings were part of the illness and not indicative of failure as a mother:

I'm not very compassionate towards myself or accepting of the fact that I was ill (rather than just being crap).

R

You haven't failed!!! The illness is making you think this way.

R

### External Stigma

External stigma comprised of the concerns many women had about how health care providers would think that they were inadequate mother if they disclosed symptoms.

Members perpetuated a strong culture of advice that urged women to contact a general practitioner, midwife or health visitor even if they did not ask for it. Half of all women who had not disclosed to a health care providers when they first posted replied that they had sought professional help following others' encouragement (n=15):

Thank you have rung the doctor. Think I just needed someone else to tell me to do it.

I

Nearly half of women who posted did not reply to say if they had taken members advice, and one woman refused to disclose to a health care provider. Women were often reluctant to disclose to their health care providers as they feared being seen as a bad mother and their baby would be taken away or social services would intervene:

I have seen the perinatal team and dr previously but kind of played down my feelings as I am scared that if I show I am not coping with my moods then they might look down on me, see me as an unfit mother and pass me over to social services.

I

Anyone got any experience with this - what did u say to the DR? What was their approach? Were you made to feel like a bad mum/mum to be? Were you strictly monitored/referred to social services after?

I

Most replies to these posts were reassuring, shared positive experiences of disclosing, and stressed getting help from a health care provider was the best course of action:

If you are seeking help and trying to sort it that's good. There's no reason to take your baby. I was honest with my gp. Nothing bad happened. I got better.

R

### Treatment Stigma

Treatment stigma was related to women’s concerns about seeking and adhering to professional treatment. It extended concepts of internal and external stigma described above.

Often posters emphasized the importance of professional help in recovery; women who started threads and posed questions were often reassured that they had *done the right thing* when they had disclosed to health care providers.

It is the people who are not seeking help and not being honest with themselves that are in the most danger, You are doing the right things, you are being objective, and seeking help.

R

Treatment was largely discussed in terms of antidepressants. Some women felt like a failure for having to take medication, which added to feelings of *weakness* for having a mental illness and being a *failure* as a mother. This stigma often centered on guilt for thinking they would harm the baby, inability to cope as a mother, and needing to rely on medication.

I still feel guilty and worried I am causing my baby harm and being selfish if I ask for drugs - did any of you guys who have taken meds struggle with this before asking?

I

Replies were embedded in a dialogue of social support and most were pro-antidepressants and encouraged women to work with health care providers. These posts often challenged stigma by promoting an alternative “good mum” discourse that challenged external and internal stigma. Posts reconceptualized what a good mother is, namely, a good mother gets help and takes treatment:

And don't worry about not being a good mum, the very fact that you posted what you did and are worried about the possible effects on the baby show that you're already a very caring mum.

R

I feel like there's a stigma attached to taking meds for a mental illness, which doesn't exist for physical illnesses...And I don't think I'm going to do DD (darling daughter) any favours by trying to prove I can be a good mum off my medication.

R

### Negative Experiences

Negative experiences with health care providers included disclosure and treatment experiences. This theme extended feelings of internal and external stigma and the majority of subsequent posts challenged stigma by promoting health care providers and treatment.

Very few posts outlined any negative experiences when women disclosed to health care providers (n=3). One woman who started a thread rebuked replies encouraging help-seeking as she had previous negative experiences with the social services and did not trust health care providers. Two women started threads to talk about bad events with health care providers:

My midwife said, and I quote, 'if you suffer from psychosis we could take your child away'. For someone feeling vulnerable this was really scary and I have not been able to relax with the pregnancy.

I

Subsequent posts condemned the midwife’s approach and said how there are good and non-judgemental healthcare providers. Replies to unhelpful health care provider experiences strongly urged women to engage with health care providers and stressed that treatment was essential for recovery.

## Discussion

### Principal Findings

This study increases our knowledge of the stigma women with perinatal mental illness may experience. In particular, it offers unique insights into how women are expressing different types of stigma on an online forum, online discourse that challenged this stigma, and the potential outcomes for help-seeking behavior. Women frequently expressed internal stigma and were concerned about external stigma from health care providers. Both were noteworthy barriers to help-seeking behavior and reply posts often challenged this stigma by sharing positive experiences of disclosure and treatment. Posts challenged some women's beliefs that health care providers would think of them as an unfit mother or social services would take their baby. Women were consistently encouraged to seek professional help.

Treatment stigma was often expressed as stigma about having a mental illness and having a mental illness as a mother. Some women felt they had failed at their role as a mother because they had to rely on medication to cope and feared treatment would harm their unborn child. Subsequent posts challenged this stigma promoting a different discourse that advocated good mothers seek help and take treatment. Many women who sought advice on whether they should disclose to a healthcare provider reported that they followed the advice of reply posts and sought help. There were 3 instances of negative experiences when disclosing symptoms to health care providers.

### Comparisons With Prior Work

Consistent with previous studies [25,43], the stigma attached to mental illness was a salient barrier to treatment and women were able to “test out” disclosing symptoms and concerns in online conversations. Like previous research, many women spoke about the stigma attached to antidepressants that contributed to a reluctance to engage in treatment [44]. This study extends our knowledge of how a forum provided an anonymous place where women could explore their understanding of perinatal mental illness and where they got encouragement to seek help and accept treatment. If women recognize that they may have a mental illness, they may be more motived to seek help like many women in this study. Women frequently expressed internal and external stigma often describing feeling like and/or being seen as a “bad mother,” an “unfit mother,” or a “failure.”

Internal, external, and treatment stigma discourse was met by replies of encouragement to get help and praised acts of disclosure to health care providers. Posts often promoted a “good mother” discourse that included the ideas that a good mother discloses and gets treatment and health care providers will not think them inadequate or take their baby away. These posts challenged the distressing dissonance between the concept of a good mother, and that of a bad mother, present in some mothers’ posts and thus reconciled that a “good mother” can have perinatal mental illness.

Unlike previous research on perinatal mental health forums, there were 3 posts that shared negative experiences of disclosing to health care providers [32-34]. Despite the vast majority of posts presenting positive experiences, the potential effect of negative posts should be considered. Some women who experience stigma can suffer with intense feelings of inadequacy and worry that health care providers will not understand or social services will become involved [20]. Future work should investigate if negative posts reinforce these anxieties and if in turn this inhibits disclosure and help-seeking behavior. The culture of discussions and attitudes to disclosure may be very different in other online communities, for example, a birth trauma support group may include many posts describing negative experiences with health care providers. This group gives women an opportunity to voice their concerns that they may not be able to do offline, however, there is potential for conversations to negatively impact healthcare decisions. Concerns are warranted, especially if the forum is unmoderated and lacks encouragement to engage with health care providers.

### Limitations

This is the first study of messages on a perinatal mental illness forum that explores stigma and disclosure and suggests potential concerns for users. Unfortunately, this study only considered one online forum as all other moderators either did not give consent or failed to reply to requests for consent. Future research should observe other forums to ensure the validity and generalizability of findings. Messages reported that half of women sought help as a direct consequence of using the forums. We do not know if there was an effect on the women who did not reply or those who read forums without posting. Users who read without posting are also known as lurkers and account for the majority of forum users [45,46]. Therefore, future research should aim to explore both posters’ and lurkers’ experiences and survey women with various levels of participation in different forums.

### Clinical Implications

This study provides further insights into the stigma women with perinatal mental illness may experience and how they communicate online. This could be used to develop targeted interventions to help women disclose to health care providers and get treatment, for example, forums could be developed to offer this support to at risk women and their subsequent disclosure could be measured against a control group. Future theoretical models could draw on this evidence and investigate if online forum use for perinatal mental illness impacts the stigma experienced by some women and if this affects disclosure to health care providers.

### Conclusions

Forum posts often expressed internal and external stigma from health care providers and treatment stigma as major barriers to disclosure and help-seeking behavior. Forum replies challenged this stigma and provided a place to discuss stigma. Forum discourse reconstructed the idea of a good mother as compatible with perinatal mental illness, especially if the woman sought help and adhered to treatment. 

The vast majority of posts encouraged women to engage with and trust in health care providers, and consequently some women sought help and engaged in treatment. This study showed that this forum has the potential to increase women's disclosure to health care providers and strengthen professional treatment uptake and adherence. However, there are possible concerns when using forums. Health care providers should exercise discernment when directing their clients to online forums.
